# Mutation spectrum of chinese amyotrophic lateral sclerosis patients with frontotemporal dementia

**DOI:** 10.1186/s13023-022-02531-2

**Published:** 2022-11-07

**Authors:** Xunzhe Yang, Xiaohai Sun, Qing Liu, Liyang Liu, Jinyue Li, Zhengyi Cai, Kang Zhang, Shuangwu Liu, Di He, Dongchao Shen, Mingsheng Liu, Liying Cui, Xue Zhang

**Affiliations:** 1 Department of Neurology, Peking Union Medical College Hospital, 100730 Beijing, China; 2 Department of Neurology, Qilu Hospital of Shandong University, 250012 Shandong, China; 3 Neuroscience Center, Chinese Academy of Medical Sciences & Peking Union Medical College, 100730 Beijing, China; 4 McKusick-Zhang Center for Genetic Medicine, Chinese Academy of Medical Sciences & Peking Union Medical College, 100730 Beijing, China; 5 Department of Neurology, Beijing Tiantan Hospital, 100070 Beijing, China

**Keywords:** Amyotrophic lateral sclerosis, Frontotemporal dementia, Gene, Variant

## Abstract

**Background:**

Studies have reported that a noncoding hexanucleotide repeat in *C9ORF72*, is the most common genetic cause of amyotrophic lateral sclerosis (ALS) and frontotemporal dementia (FTD) among Caucasian population, nevertheless it is rare in Chinese population. Therefore, we aimed to investigate the mutation spectrum of Chinese ALS patients with FTD (ALS-FTD).

**Methods:**

ALS patients with and without cognitive impairments were enrolled. Clinical features were collected including age, sex, disease duration, ALSFRS-r, family history and cognitive evaluation. Thirty-six ALS genes were screened by whole exome sequencing (WES) and repeat-primed polymerase chain reaction (PCR) were used for detection of and abnormal repeat expansions of *C9ORF72*.

**Results:**

A total of 1208 patients, including 66 familial ALS (FALS) and 1142 sporadic ALS (SALS) patients were included. Twenty-three patients with sporadic ALS and one familial ALS index had concomitant FTD, which accounts for 1.99% (24/1208) of patients with ALS. In sporadic ALS-FTD, one case harboring *C9ORF72* expansion variant, two cases harboring *ANXA11* variants and one individual carrying *CCNF* variant were identified. A recurrent *UBQLN2* variant was detected in a familial ALS-FTD patient. All of the ALS-FTD patients carrying variants in known causative genes manifested motor symptom onset (two bulbar onset and three limb onset) and developed cognitive impairment thereafter. It is not easy to draw a conclusion of the genotype-phenotype association in ALS-FTD with certain variants, limited by the small number of patients.

**Conclusion:**

Our findings provide an overview of spectrum of genetic variants in Chinese ALS-FTD patients. Variants of uncertain significance in *UBQLN2, ANXA11* and *CCNF* were identified and further studies are required for causal relations of these variants with ALS-FTD.

**Supplementary information:**

The online version contains supplementary material available at 10.1186/s13023-022-02531-2.

## Background

Amyotrophic lateral sclerosis (ALS) is a relentlessly progressive neurologic disease characterized by progressive paralysis and ultimately respiratory failure, with an incidence of 2.6/100 000 per year [1]. Familial ALS accounts for 5 to 10% of all ALS cases, with mutations in several genes including *SOD1*, *TARDBP*, *OPTN*, *FUS*, *UBQLN2* and *VCP*. Frontotemporal dementia (FTD) is the second most common cause of early onset dementia, and is characterized by progressive degeneration of the frontal and temporal cortex. It occurs with an incidence of 3.5–4.1/100,000 per year in individuals under 65 [2]. A family history of dementia or psychiatric conditions is present in 40% of FTD patients. Mutations in *MAPT* and *GRN* and a hexanucleotide expansion in *C9ORF72* together accounts for approximately 15% of familial FTD cohorts; mutations in other genes, including *VCP*, *CHMP2B*, and *TBK1*, are rare [3, 4].

ALS and FTD were traditionally considered as completely different neurological disorders with distinct clinical features. Studies over the last 30 years have demonstrated the co-occurrence of ALS and FTD. About 15% of ALS cases showing a clear diagnosis of FTD while around 15% of patients with FTD displaying ALS [5, 6]. What’s more, the description of several mutations that are common to both ALS and FTD provides a genetic link between them. Studies have reported that a noncoding hexanucleotide repeat in *C9ORF72*, is the most common genetic cause of ALS and FTD among Caucasian population [7, 8]. Mutations in *UBQLN2* [9, 10], *VCP* [11], *TARDBP* [12], *FUS* [13] have also been identified in patients with ALS and FTD. Thus, ALS and FTD are now considered to be a spectrum of one overlapping diseases, commonly referred to as ALS/FTD.

Mutation spectrum of ALS varies among populations of different ethnic background. While *C9ORF72* is the most prevalent mutant gene in Caucasian, it is rare in Chinese population. Few studies had explored the genetic epidemiology of ALS/FTD in Chinese populations. Therefore, we aimed to investigate the mutant condition in Chinese ALS patients with FTD (ALS-FTD) and to characterize the genotype-phenotype association of these patients.

## Methods

### Participants and data collection

This study included 1208 patients who were diagnosed of definite or probable ALS according to revised E1 Escorial criteria at neurology department of Peking Union Medical College Hospital (PUMCH) from September 2011 to September 2021. All patients were registered into the ALS clinical research database registration system of PUMCH and a series of clinical features were collected including age, sex, disease duration, diagnosis delay, onset site, disease evolution, revised ALS functional rating scale (ALSFRS-r), family history and cognitive evaluation. A positive family history was defined as relatives within two generations were diagnosed of ALS. Cognitive evaluation battery included: Mini-Mental State Examination score (MMSE) and the Montreal Cognitive Assessment (MoCA) and Edinburgh Cognitive and Behavioural ALS Screen (ECAS) [14]. Diagnosis of ALS with behavioral variant FTD required the individual met at least one non-overlapping supportive diagnostic features from the Neary criteria [15] for FTD. Besides, 2445 ethnicity matched healthy controls were also included in this study.

### Genetic testing and bioinformation analysis

Peripheral venous blood samples were collected from the ALS patients, and DNA was isolated from mononuclear cells using a DNA isolation kit (Blood DNA Kit V2, CW2553), according to the ALS online database and previous reports [16–18]. Thirty-six genes identified in ALS-FTD, ALS, and FTD were screened by whole exome sequencing (WES), including the genes *SOD1, ALS2, SETX, FUS, VAPB, TARDBP, OPTN, VCP, UBQLN2, SIGMAR1, FIG4, CHMP2B, PFN1, NEFH, PRPH, TFG, TAF15, GRN, CHCHD10, TUBA4A, TBK1, NEK1, GLE1, MATR3, CCNF, ANXA11, HNRNPA1, SQSTM1, ERBB4, TIA1, SPG11*, *KIF5A, GLT8D1, DNAJC7, TBP, CTSF and MFSD8*. In brief, WES procedure comprised three standard steps: end-repair of fragmented DNA, A-tailing, and adapter ligation and amplification. Sequencing was completed by the HiSeq2000 platform (Illumina, San Diego, California, USA) as paired-end 200 bp reads. Variants with read depth ≥ 10 and genotype quality ≥ 20 were retained. Average depth and coverage were above 100X and 98.9%, respectively. The sequence was aligned to the human reference genome (UCSC hg19) using a Burrows-Wheeler Aligner and then reformatted using SAM tools (10.1093/bioinformatics/btv178). Variant frequencies were determined in gnomeAD (http://gnomad-old.broadinstitute.org/), ExAC (http://exac.broadi-nstitute.org/) and 1000 Genomes (http://www.1000genomes.org) to remove the common single nucleotide polymorphisms (SNP). Only non-synonymous, splicing and frameshift variants with a minor allele frequency (MAF) of less than 0.5% or absent in population databases were selected for further analysis. SIFT (http://sift.bii.a-star.edu.sg/) and PolyPhen2 (http://genetics.bwh.harvard.edu/pph2/) were used for variant annotation. Besides, fragment-length and repeat-primed PCR was performed to detect duplications or deletions of *ATXN2* and *C9ORF72* in ALS/FTD patients. The criteria used to detect causative mutations were based on the recommendations of the American College of Medical Genetics and Genomics (ACMG) [19].

### Verification of variants by Sanger sequencing

PCR has been performed to validate the variants identified by WES. Primers were designed using Primer 3 online software (http://bioinfo.ut.ee/primer3-0.4.0/). Amplification products were subjected to Sanger sequencing using the ABI 3730 automated DNA-sequencing system (Applied Biosystems). The sequence was aligned to the human reference genome (UCSC hg19) using CodonCode Aligner tool.

### Ethics

The study was approved by the ethics committee of Peking Union Medical College Hospital. Informed consent forms were obtained from all patients or their families.

## Results

### Clinical information of ALS patients

A total of 1208 patients, including 66 FALS probands and 1142 unrelated SALS patients were enrolled in current study. The male/female ratios were 1.7:1.0 (15/9). Of which, 23 SALS patients and 1 FALS index had concomitant frontotemporal lobar dementia, which accounts for 2.01% (23/1142) ALS-FTD in SALS and 1.52% (1/66) in FALS respectively. Eleven patients had bulbar-onset while fourteen patients had limb-onset. Of 24 ALS-FTD patients, nine presented motor symptoms onset, 11 presented cognitive impairment onset and four patients presented motor and cognitive deficits almost simultaneously. Cognition deteriorated in all patients over disease progression. The demographic features of patient are presented in Table 1.

**Table 1:** Demographics of patient cohort

Variable	Total ALS	ALS-FTD
n	1208	24
Male, n (%)	706(58.4)	15(62.5)
Onset age (years)	49.3 ± 11.4	60.1 ± 11.4
Bulbar onset, n (%)	170(14.1)	9(37.5)
Family history, n (%)	66(5.5)	1(4.2)

*ALS* Amyotrophic lateral sclerosis, *FTD* Frontotemporal dementia, *ALS-FTD* ALS patients with FTD

### Genetic results

In total, one *C9ORF72* expansion variant were identified in one sporadic ALS-FTD patient. The same missense variants c.107 C > G (p.P36R) in the ANXA11 (NM_145869.2) was found in two unrelated ALS-FTD patients. One heterozygous missense variant c.499G > A (p.V167M) in the *CC*NF (NM_001761.3) was identified in a sporadic ALS-FTD patient. A heterozygous variant c.1498 C > T (p.P500S) in the UBQLN2 (NM_013444.3) were identified in one familial ALS-FTD patient (Table 2). None of these was detected in 2445 healthy control individuals.

**Table 2:** Genetic profile of five patients identified in our study

ID	Gene	Transcript ID	Genomic location	Exon	Nucleoid Changes	Amino acid change	gnomAD	ExAC	Conservation GERP++	SIFT	Polyphen2	ACMG
P474	*ANXA11*	NM_145869.2	chr10-80170864	Exon5	c.107 C > G	p.P36R	0	0.00090%	4.7	0.018	0.994	VUS
P919	*ANXA11*	NM_145869.2	chr10-80170864	Exon5	c.107 C > G	p.P36R	0	0.00090%	4.7	0.018	0.994	VUS
P982	*UBQLN2*	NM_013444.4	chrX-56,565,371	Exon1	c.1498 C > T	p.P500S	NA	NA	4.18	0.342	0.118	VUS
P478	*CCNF*	NM_001761.3	chr16-2437281	Exon5	c.499G > A	p.V167M	0	0.0018%	5.68	0.069	0.4	VUS
P623	C9orf72 expansion	-	-	-	-	-	-	-	-	-	-	Pathogenic

*ACMG* the American College of Medical Genetics and Genomics, *NA* not applicable

Based on ACMG criteria, the three detected variants were categorized as variant of uncertain significance (VUS). Genetic variants account for 12.5% (3/24) SALS-FTD patients and 100.0% (1/1) FALS-FTD. The average sequencing depth of coverage ranged from 88.2 to 142.6, with > 99% sequences mapping to the genome.

One recurrent missense variant in *UBQLN2*, c.1498 C > T (p.P500S) were identified in one familial ALS/FTD patient. The same genetic alteration had already been reported in ALS by Teyssou et al. [20]. This variant was absent from the exome variant server including the ExAC, gnomAD and the 1000 Genomes Project, as well as our inhouse controls.

Novel heterozygous variant in *ANXA11*, c.107 C > G (p.P36R) was identified in two unrelated sporadic ALS-FTD patients. The p.P36R variant was predicted to be conserved through revolution. The variant was predicted by to be ‘damaging’ by SIFT and ‘probably damaging’ by Polyphen-2 in silico analysis.

Additionally, another missense variant in *CCNF*, c.499G > A (p.V167M) was identified in one sporadic ALS-FTD patient. The p.V167M variant was highly conserved among different species. The p.V167M variant is predicted to be ‘tolerable’ by SIFT and ‘probably damaging’ by Polyphen-2 in silico analysis.

The patients mentioned above were negative for other common ALS causal gene mutations, including *ATXN2* abnormal repeat expansions.

### Clinical characteristics of patients with certain variants

All of the ALS-FTD patients carrying variants in known causative genes manifested motor symptom onset (two bulbar onset and three limb onset) and developed cognitive impairment thereafter. It is not easy to draw a conclusion of the genotype-phenotype association in ALS-FTD with certain variants, limited by the small number of patients. An additional file shows the clinical information of patients in more detail [see Additional file 1].

## Discussion

Advances in sequencing technologies have shed fresh insight into the concept of ALS and FTD from two separate clinical entities into a continual disease spectrum. Identification of the genes related to ALS/FTD spectrum may facilitate our understanding of the genetic etiology and common pathogenic factors leading to neurodegeneration. In total, four nonsynonymous variants in *UBQLN2*, *ANXA11* and *CCNF*, as well as one *C9ORF72* expansion variant, were identified in 5 unrelated ALS-FTD patients.

Historically, comorbidity rates of FTD were documented as 3% in sporadic ALS and 15% in familial ALS [21], but recent studies [2, 6, 22] using frontal lobe–based neuropsychological measures report the vary from 28 to 48%. The comorbidity rates of FTD in SALS (2.01%) and in FALS (1.52%) in our database, which is lower than that in the Caucasian population. The difference may be owing to not only different ethnicity and diagnostic criteria, but also the limits of health care and economy in some parts of China.

Our study revealed the discrepancy of mutation spectrum between Chinese and Caucasians. *C9ORF72* repeat expansion is the most common mutation in studies of pan-European and North American patient populations [23, 24], which is not as common among Chinese, Korean, and Japanese ALS patients [25–27]. In our previous study, 40.0% (8/20) of FALS and 18.8% (44/234) of SALS carried mutations in these genes, with a mutant frequency varying from *SOD1* (FALS 30.6%, SALS 1.5%) to *UBQLN2* (FALS 5%, SALS 0) [28]. One *C9ORF72* repeat expansion was identified in a 65-year-old female with limb-onset ALS, with a mutant frequency of 0.39% (1/254), which is consistent with low frequency of *C9ORF72* mutation as previous reported. In the ALS-FTD cohort, only one *C9ORF72* repeat expansion was found. Recurrent *UBQLN2* variant was found in one FALS-FTD patient with a frequency of 4.0% (1/24). *ATXN2* repeat expansion and variants in VCP, TARDBP, FUS was not detected in current ALS-FTD patients.

ALS-linked variants in UBQLN2 were found to be associated with dysfunction of autophagy, neuroinflammation and formation of stress granules. These recent data surely placed *UBQLN2* as an essential player in noxious protein accumulation and clearance pathways in ALS and FTD pathogenesis. The p.P500S variant in *UBQLN2* was previously reported in a female European patient with ALS and had a common European founder. Weakness onset appeared in the right arm at 62 years of age. Her bulbar function rapidly worsened and she died from respiratory failure after 16 months of disease duration with no cognitive defect. In the current study, the woman with the p.P500S variant had a bulbar onset ALS at 63 years of age with cognitive impairment. She died from respiratory failure after 24 months of disease duration. It is difficult to pinpoint common features of *UBQLN2*-mutated patients in these one studies.

Annexin 11 encoded by *ANXA11* is a member of the annexin protein family. ANXA11 protein attaches to RNP granules via its structurally disordered N-terminal domain, and to lysosomes via the C-terminal annexin repeats. The dual biophysical properties of ANXA11 protein allow it to act as a molecular tether that binds neuronal stress granules (and possibly other RNP granules) to lysosomes [29]. It is speculated that ALS-associated missense mutations in either the N-terminal LCD or in the annexin repeat domain of ANXA11 disrupt formation of the molecular tether and are associated with impaired delivery of mRNA to axon terminals for local protein synthesis [29]. However, the pathogenetic mechanism of *ANXA11* variant is under investigation.

Smith et al. [30] screened 751 familial ALS patient by whole-exome sequences and identified six variants including a nonsynonymous variant p.D40G and p.D38G in *ANXA11* in 13 individuals. The p.D40G mutation segregated with disease in one kindreds and was present in another two unrelated cases. They observed a unique feature of abundant Annexin A11–positive protein aggregates in spinal cord motor neurons and hippocampal neuronal axons of ALS patient carrying the p.D40G variant, along with classical pathological features of ALS. The c.107 C > G (p.P36R) variant is presented once in the ExAC East Asian population (MAF 1⁄4 0.00012). The p.P36R variant is located in the CACY binding site in the N-terminal of annexin A11 near the p. D38G and p.D40G variant and most bioinformatic tools indicate pathogenicity.

CCNF is the substrate-recognizing component of the Skp1-cullin-F-box E3 ubiquitin-ligase complex, which is responsible for tagging proteins with ubiquitin and marking them for degradation via the ubiquitin-proteasome system [31]. Neuronal cells overexpressing mutant CCNF show an increase in ubiquitin-tagged proteins, which include TDP43. *CCNF* was identified as a causative gene for ALS on the basis of exome sequence analysis [18]of a large family of European descent who had ALS, frontotemporal dementia, or both diseases, with an autosomal dominant pattern of inheritance. The authors reported additional, potentially pathogenic variants in *CCNF* in familial ALS/FTD cases with an overall mutation frequency that ranged between 0.6 and 3.3% in white populations [31]. In current study, a missense variant in *CCNF*, c.499G > A(V167M) was identified with a frequency of 4.0% (1/24) among ALS-FTD patients.

## Conclusion

In summary, ALS-FTD patients were rare in ALS cohort. Our study provided an overview of mutation spectrum of genetic variants in Chinese ALS-FTD patients. In sporadic ALS-FTD, one case harboring *C9ORF72* expansion variant, two cases harboring *ANXA11* variants and one individual carrying *CCNF* variant were identified. A recurrent *UBQLN2* variant was detected in a familial ALS-FTD patient. The results uncovered the discrepancy of mutation spectrum between Chinese and Caucasians. Variants in *UBQLN2*, *ANXA11* and *CCNF* were identified and further studies are required for causal relations of these variants with ALS-FTD.

## Electronic supplementary material

Below is the link to the electronic supplementary material.

Supplementary Material 1

## Data Availability

All data generated and/or analysed during the current study are not publicly available due to personal information of the patient, but available from the corresponding author on reasonable request.

## Declarations

Ethic approval and consent to participate

The study was approved by the ethics committee of Peking Union Medical College Hospital. Informed consent forms were obtained from all patients or their families.

## Abbreviations

ALS: Amyotrophic lateral sclerosis.
FTD: Frontotemporal dementia.
ALS-FTD: ALS patients with FTD.
FALS: familial ALS.
SALS: sporadic ALS.
WES: whole exome sequencing.
PCR: polymerase chain reaction.
PUMCH: Peking Union Medical College Hospital.
MMSE: Mental State Examination score.
MoCA: Montreal Cognitive Assessment.
ECAS: Edinburgh Cognitive and Behavioural ALS Screen.
ALSFRS-r: ALS functional rating scale-revised.
MAF: minor allele frequency.
ACMG: the American College of Medical Genetics and Genomics.
