# Supplementary material for: Mutation spectrum of chinese amyotrophic lateral sclerosis patients with frontotemporal dementia
Source: Orphanet J Rare Dis. 2022 Nov 7;17:404. doi: 10.1186/s13023-022-02531-2 (PMC9641840; doi:10.1186/s13023-022-02531-2)
Supplement: Supplementary file 1 — Supplementary Material 1 [file 13023_2022_2531_MOESM1_ESM.docx]

**Clinical characteristics of patient with the *UBQLN2* variant**

The woman with the p.P500S variant was a bulbar onset ALS at 63 years of age. Four months later, muscle weakness was followed in the right leg and 6 months later the arms. Pyramidal signs were all positive on physical examination. Electromyography showed signs of lower motor neuron involvement in 4 limbs and bulbar muscles. Her MMSE was 21/30 while MoCA test score was 18/30. She died from respiratory failure after 24 months of disease duration. Her only brother developed a bulbar onset ALS at 60 years (dysarthria and phonation) and was dead earlier than her with a disease duration of 28 months. Their father died at 61 years from hepatic cirrhosis and her mother died at 84 years from trauma and bone fracture. Therefore, the evidence of segregation with disease in this small family was unavailable.

**Clinical characteristics of patients with *C9ORF72* repeat expansion mutation**

The man with *C9ORF72* repeat expansion was a limb onset ALS around 56 years of age. Eight months later, he presented a loss of empathy and simple repetitive movements (turn on an off the lights). Pyramidal signs were positive while EMG demonstrated chronic and acute denervation in three body segments. His MMSE was 22/30 while MoCA test score was 17/30. He died from respiratory failure at 58 years following 24 months of disease duration.

**Clinical characteristics of patients with the *ANXA11* variant**

One of the p.P36R variant carrier initially presented slurred speech at age 70 years. A few months later, he developed muscle weakness and atrophy in lower limbs. About a year and a half later, His family members reported that he became easily irritable, aggressive and inappropriate behaviors, such as laughing at inappropriate occasions. Over the subsequent one year, his both arms showed weakness. EMG demonstrated chronic and acute denervation changes in the cervical, thoracic, and lumbar segments. MRI showed bilateral temporal lobe atrophy and moderate frontal atrophy. 18F-fluorodeoxyglucose-PET imaging showed bilateral frontotemporal hypometabolism. His Mini-Mental State Examination score (MMSE) was 20/30, and the Montreal Cognitive Assessment (MoCA) test score was 15/30. He was diagnosed as having ALS with behavioral variant FTD (bvFTD). Another case with p.P36R variant presented with muscular weakness accompanied by cognitive impairment. The detailed clinical information is summarized in table 2. The two cases have been previously reported in another study of our team[21].

**Clinical characteristics of patients with the *CCNF* variant**

The man with the p.V167M variant had upper limb weakness at 68 years of age. Half a year later, fasciculations were observed in upper limbs and tongue. Pyramidal signs including bilateral Babinski and Hoffmann signs appeared. EMG demonstrated chronic and acute denervation in four body segments. His MMSE was 23/30 while MoCA test score was 19/30. bvFTD were also diagnosed at that time. He died from respiratory failure at 70 years following 11 months of disease duration.
